# Transitions to Long-Term Residential Care Settings

**DOI:** 10.1093/geroni/igab046.855

**Published:** 2021-12-17

**Authors:** Bram de Boer, Hilde Verbeek, Joseph Gaugler

## Abstract

During their life course, many older adults encounter a transition between care settings, for example, a permanent move into long-term residential care. This care transition is a complex and often fragmented process, which is associated with an increased risk of negative health outcomes, rehospitalisation, and even mortality. Therefore, care transitions should be avoided where possible and the process for necessary transitions should be optimised to ensure continuity of care. Transitional care is therefore a key research topic. The TRANS-SENIOR European Joint Doctorate (EJD) network builds capacity for tackling a major challenge facing European long-term care systems: the need to improve care for an increasing number of care-dependent older adults by avoiding unnecessary transitions and optimising necessary care transitions. During this symposium, four presenters from the Netherlands and Switzerland will present different aspects of transitions into long-term residential care. The first speaker presents the results of a co-creation approach in developing an intervention aimed at preventing unnecessary care transitions. The second speaker presents an overview of interventions aiming to improve a transition from home to a nursing home, highlighting the clear mismatch between theory and practice. The third speaker presents the impact of the COVID-19 pandemic on transitions into long-term residential care using an ethnographic study in a long-term residential care facility in Switzerland. The final speaker discusses the results of a recent Delphi study on key factors influencing implementing innovations in transitional care. The discussant will relate previous findings on transitional care with a U.S. perspective.

